# Metformin Increases Serum Isthmin-1 Levels and Lowers Low-Density Lipoprotein: Potential Implications for Lipid Metabolism in T2DM

**DOI:** 10.3390/medicina61030522

**Published:** 2025-03-17

**Authors:** Merve Yilmaz Bozoglan, Tuncay Kuloglu, Nevzat Gozel, Faruk Kılınc, Erkan Cakmak, Ramazan Fazıl Akkoç, Betül Dağoğlu Hark

**Affiliations:** 1 School of Medicine, Department of Medical Pharmacology, Firat University, Elazig 23200, Türkiye; 2 School of Medicine, Department of Histology and Embryology, Firat University, Elazig 23200, Türkiye; tkuloglu@firat.edu.tr; 3 School of Medicine, Department of Internal Medicine, Firat University, Elazig 23200, Türkiye; ngozel@firat.edu.tr; 4 School of Medicine, Department of Endocrinology, Firat University, Elazig 23200, Türkiye; fkilinc@firat.edu.tr; 5 School of Medicine, Department of Intensive Care, Firat University, Elazig 23200, Türkiye; e.cakmak@firat.edu.tr; 6 School of Medicine, Department of Anatomy, Firat University, Elazig 23200, Türkiye; ramazan_fazil@hotmail.com; 7 School of Medicine, Department of Biostatistics, Firat University, Elazig 23200, Türkiye; bdagoglu@firat.edu.tr

**Keywords:** adipokine, isthmin 1, metformin, T2DM, LDL

## Abstract

*Background and Objectives:* Type 2 Diabetes Mellitus (T2DM) is a metabolic disease caused by the failure of the skeletal muscle, liver and adipose tissue to respond to insulin. Metformin is the first choice for the treatment of T2DM. Isthmin 1 (Ism1) is a newly discovered adipokine that affects all carbohydrate, lipid and protein metabolism. This study examines the changes in serum and salivary levels of Ism1 in patients using metformin, considering its potential as a follow-up marker for T2DM if present in the salivary glands. *Materials and Methods:* The study included 30 newly diagnosed T2DM patients and 30 non-diabetic controls. Ism1 was measured by ELISA in serum and saliva after 3 months and compared with routine biochemical parameters. Immunostaining of Ism1 was performed in salivary glands. *Results:* Ism1 was immunohistochemically detected in salivary glands for the first time. Serum Ism1 levels increased significantly after 3 months of metformin treatment (*p* = 0.028). The increase in salivary Ism1 levels did not reach statistical significance. Fasting plasma glucose (FPG) (*p* < 0.001), HbA1c (*p* < 0.001) and LDL (*p* = 0.015) levels decreased with metformin. There was a significant negative correlation between the increase in Ism1 levels and the decrease in LDL levels (rho = −0.362, *p* = 0.05). *Conclusions:* Despite its first detection in salivary glands, the hypothesis that Ism1 may be a surveillance marker in T2DM could not be confirmed. The negative correlation of Ism1 with LDL levels suggests that Ism1 may contribute to the ameliorative effect of metformin on serum lipids. Further studies are needed to support this conclusion.

## 1. Introduction

Type 2 Diabetes Mellitus (T2DM) is a metabolic disease characterized by insulin resistance and high plasma glucose levels, which occur when the skeletal muscle, liver and adipose tissue cells fail to respond properly to insulin. Glucose uptake is impaired, and cells are unable to utilize glucose properly. Metformin remains recognized as the first-line and primary therapeutic agent for treating T2DM in the absence of contraindications. However, metformin also has an important role in the treatment of insulin resistance, metabolic syndrome and polycystic ovary syndrome [1]. The common mechanism of the action of metformin in these pathologies is its ability to reduce hepatic glucose output and enhance glucose uptake in the peripheral tissues. In addition to its effects on glucose metabolism, metformin has been shown to improve lipid parameters, inflammatory biomarkers, cardiovascular events and neurodegenerative diseases, and it has even demonstrated chemotherapeutic activity in certain cancers [2,3,4]. Recent studies, such as the Metformin in Longevity Study, have highlighted the potential effects of metformin on delaying aging and increasing life expectancy [5]. It is unclear whether all these effects of metformin are due to its effects on glucose homeostasis or to other events at the cellular level, including the inhibition of the mammalian target of rapamycin (mTOR) [6].

Isthmin-1 (Ism1) was first discovered as a prominently secreted protein in the isthmus of Xenopus embryos [7]. Ism1 is distributed throughout the body, particularly in the brain and lungs. Ism1 is also found in the eye, kidney, skeletal muscle and heart [8]. In later studies, Heeren et al. [9] found that Ism1 is an adipokine secreted by adipocytes and has insulin-like effects. Further studies by Jiang et al. [10] revealed that pharmacological doses of Ism1 increased glucose uptake independent of insulin. In addition to its effects on carbohydrate metabolism, the same study also revealed the effects of Ism1 on fat metabolism by inhibiting lipogenesis and its impact on protein metabolism by stimulating protein synthesis in the liver [10]. The multifaceted nature of Ism1, affecting various metabolic pathways, suggests it may possess additional biological activities. Consequently, Ism1 has been identified as a member of a protein family that plays roles in angiogenesis, immunity, growth, and craniofacial development [11,12]. In addition to being a multifunctional molecule, Ism1—as outlined above—is especially notable for its effects on metabolism. Its suppression of hepatic lipogenesis may even suggest that it is superior to insulin, the body’s only hypoglycemic hormone. Ism1 acts on carbohydrate metabolism by regulating glucose uptake through activation of the mTOR-phosphatidylinositol 3-kinase (PI3K) pathway [10]. Elevated levels of Ism1 are associated with a reduced risk of diabetes [13]. Although there are many options for the treatment of diabetes today, the process, complications and consequences of diabetes remain unresolved for patients, physicians and the health economy. Therefore, it is vital to understand the molecular mechanisms and potential underlying mediating biomarkers in the pathology and treatment of T2DM. There is an increasing number of studies reporting the effects of newly discovered adipokines on metabolism [14]. However, there are molecules that have not yet been discovered or are not fully understood. Ism1 is one of these molecules. There is only one study comparing the long-term therapeutic effect of Ism1 with metformin. In this study, the separate and combined effects of metformin and Ism1 were observed in diet-induced obese mice. The 21-day treatment did not significantly change BMI, either alone or in combination. On the other hand, FPG decreased in all groups compared to the untreated group. Insulin-sensitizing effects were observed mostly in the combined Ism1 and metformin group [10]. To our knowledge, there is no human study evaluating Ism1 and metformin, which use the same mTOR action pathway, together. This study aims to examine the changes in serum and salivary levels of Ism1 in patients using metformin, considering its potential as a follow-up marker for T2DM if present in the salivary glands.

## 2. Materials and Methods

### 2.1. Study Participants and Study Plan

**Figure 1:**
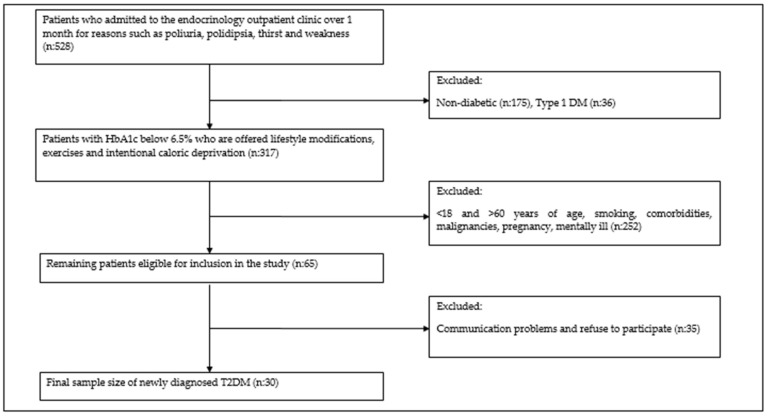
Flow chart suggesting the number of participants included and excluded.

The study was conducted in accordance with the Declaration of Helsinki and approved by the Non-Interventional Research Ethics Committee of the Firat University (protocol code: 2022/12-21 and date of approval: 20 January 2022. A total of thirty patients with newly diagnosed T2DM according to the American Diabetes Association criteria who met the inclusion criteria were enrolled in the study, based on the number of participants in another previous study by the authors of our article [15]. The inclusion criteria of the participants were determined as follows: 18–60 years of age, non-smoking, no comorbidities or malignancies and being mentally healthy (Figure 1). An equal number of healthy volunteers were randomly selected. Written informed consent was obtained from each participant. Thirty newly diagnosed T2DM patients and thirty healthy controls had venous blood drawn after an eight-hour fast. Simultaneously, unstimulated saliva was collected after the participants rinsed their mouths with water, and then they expelled any residue. To develop a tolerance to the gastrointestinal effects of metformin, patients were trained to take one gram of oral metformin daily, one half of a tablet in the morning and evening on a full stomach for the first week. Following this initial week, patients took two grams of metformin, one gram each morning and evening, for 12 weeks. During this period, no special dietary program was determined for the patients. At the end of the twelve weeks, unstimulated saliva and blood were again collected from all participants to compare the change in Ism1 and other laboratory values. Demographic information (age, gender) and laboratory parameters (biochemistry and complete blood count) were obtained from the university hospital records. Among these, hemoglobin A1c (HbA1c), low-density lipoprotein cholesterol (LDL) and triglycerides (TG) were selected as the parameters to be used in statistical analyses as they were considered relevant to the study design.

### 2.2. Biochemical Measurement of Isthmin-1 and Other Parameters

Biochemical analyses were conducted using the enzyme-linked immunosorbent assay (ELISA) technique. Serum samples obtained after the centrifugation of whole blood and saliva samples of the patients were stored at −80 °C (Nuaire, Mexico). On the measurement day, a human isthmin 1 kit (Sunred Biotechnology Company, Shanghai, China, Ref. DZE201124050 and Catalog no: 201-12-4050) was prepared according to the instructions. ELISA measurements were carried out in accordance with the instructions in the catalog of the kit. The samples were assayed by using a plate reader (Multiskan FC, Thermo Fisher Scientific, Waltham, MA, USA). The measurement range of the Ism1 ELISA kit was 2.5 to 80 ng/mL, and the minimum measurable level (sensitivity) was 0.357 ng/mL. Additionally, the kit had an intra-assay value of <8% and an inter-assay Cv value of <11%. Routine biochemical parameters other than HbA1c were measured with an Olympus AU 600 (Olympus Optical Co., Ltd., Tokyo, Japan) automatic analyzer using Olympus kits. For measuring HbA1c levels, the Bio-Rad Variant II, TUV Rheinland of North America, Inc. US., L70200401 (Austin, TX, USA) device was used.

### 2.3. Immunostaining

Submandibular and parotid glands that were ensured from the university archives of pathology were used for Isthmin-1 immunoreactivity. Tissue samples were stored in the archive at room temperature after formalin fixation and paraffin embedding, which preserves the tissue intact and enables long-term storage. Approximately 5–6 mm thin sections were taken on polylysine slides. Deparaffinized tissues were passed through graded alcohol series and boiled in citrate buffer solution at pH:6 in a microwave oven (750 W) for 15 min for antigen retrieval. Tissues were allowed to cool at room temperature for approximately 20 min after boiling. Then, after washing with PBS (Phosphate-Buffered Saline, P4417, Sigma-Aldrich, Waltham, MA, USA) for 3 × 5 min, it was incubated with hydrogen peroxide block solution for 5 min to prevent endogenous peroxidase activity (Hydrogen Peroxide Block, TA-125-HP, Lab Vision Corporation, Fremont, CA, USA). Ultra V Block (TA–125-UB, Lab Vision Corporation, Fremont, CA, USA) solution was subsequently applied to the tissues that had been washed with PBS for 5 min to block the background dye. Afterwards, the tissues were incubated with the isthmin-1 primary antibody (ISM1 Antibody, PA5-24968, Invitrogen, Waltham, MA, USA) that was diluted 1/200 for 60 min at room temperature in a humid environment. Tissues washed again with PBS for 3 × 5 min were treated with a secondary antibody (biotinylated Goat Anti-Polyvalent (anti-mouse/rabbit IgG), TP–125-BN, Lab Vision Corporation, Fremont, CA, USA) for 30 min at room temperature in a humid environment. Tissues washed with PBS again were incubated with Streptavidin Peroxidase (TS–125-HR, Lab Vision Corporation, Fremont, CA, USA) for 30 min in a humid environment at room temperature and they were taken into PBS. DAB (3,3′-Diaminobenzidine) chromogen was dripped onto the tissues, and after the image signal was obtained under the light microscope, they were simultaneously washed with PBS. Tissues that were counterstained with Harris hematoxylin were washed with PBS and distilled water and covered with an appropriate closure solution. The prepared preparations were examined and photographed under a Leica DM500 microscope (Leica DFC295, Leica Microsystems, Wetzlar, Germany).

### 2.4. Data Analysis

The statistical software package of IBM SPSS Statistics Version 22.0 was preferred to analyze the data. Numbers and percentages were used to describe categorical variables, while continuous variables were summarized using mean and standard deviation (SD) or median and minimum–maximum values, depending on their distribution. The distributional assumptions of all continuous variables were evaluated using the Shapiro–Wilk test for normality and Levene’s test for homogeneity of variances. Categorical variables between the control group and new-onset DM were compared using the Chi-square test. For numerical measurements, an Independent Samples T-Test was applied when the assumptions of normality and homogeneity of variance were met. In cases where these assumptions were violated, the Wilcoxon Signed Rank Test was employed to compare the laboratory values of newly diagnosed diabetic patients with their laboratory values after three months of metformin treatment. Since some numerical measurements did not adhere to a normal distribution, the correlation between these continuous measurements was analyzed using Spearman’s rank correlation analysis. Spearman’s correlation analysis determines whether an increase or decrease in one variable increases or decreases the other, even if there is no linear relationship between the data. In addition, data with extreme outliers can also be evaluated with this analysis since it does not require conformity to a normal distribution. One of the graphs used to show the results of Spearman correlation analysis is the scatter plot. This shows that if there is a positive correlation in the data, the points generally trend upwards, whereas if there is a negative correlation, the points tend to trend downwards. In this study, the correlation between Ism1 and FPG, HbA1c and LDL measurements, which change at the same time, was analyzed by Spearman correlation and shown by Scatter plot. The statistical significance level was set at 0.05 for all analyses.

## 3. Results

### 3.1. Immunreaktivity of Isthmin-1

**Figure 2:**
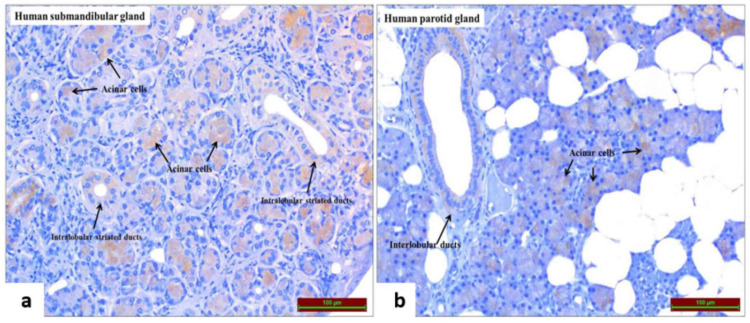
Ism1 immunoreactivity of the diabetic patients in intralobular striated ducts and acinar cells in the submandibular gland (**a**) and in the interlobular ducts and acinar cells in the parotid land (**b**).

Ism1 immunoreactivity was detected in intralobular striated ducts and acinar cells in the submandibular gland (Figure 2a) and in interlobular ducts and acinar cells in the parotid gland (Figure 2b). The mean age of patients with newly diagnosed diabetes on metformin treatment was higher than in the control group (*p* = 0.004). In addition, most participants were male in both the control group and the group with T2DM (Table 1).

### 3.2. Changes in Biochemical Parameters During Metformin Treatment

Serum samples of patients in the T2DM group were tested twice, once before and once after 12 weeks of metformin treatment. FPG, HbA1c and LDL levels were increased in T2DM patients at diagnosis (*p* < 0.001, *p* < 0.001 and *p* = 0.004, respectively). After 12 weeks of metformin treatment, FPG, HbA1c and LDL decreased significantly (*p* < 0.001, *p* < 0.001 and *p* = 0.015, respectively). Triglyceride (TG) levels decreased borderline insignificantly after treatment (*p* = 0.054) (Table 2).

### 3.3. Changes in Isthmin 1 Levels During Metformin Treatment

Serum Ism1 levels were almost the same in the control group and patients with newly diagnosed diabetes (*p* = 0.923) (Table 3). On the other hand, Ism1 levels increased significantly in people with diabetes receiving metformin treatment at the end of 12 weeks (*p* = 0.028). Salivary Ism1 levels also increased with metformin treatment in diabetic patients. However, this increase did not reach statistical significance (Table 3).

### 3.4. Analysis of Correlation Between Parameters

**Figure 3:**
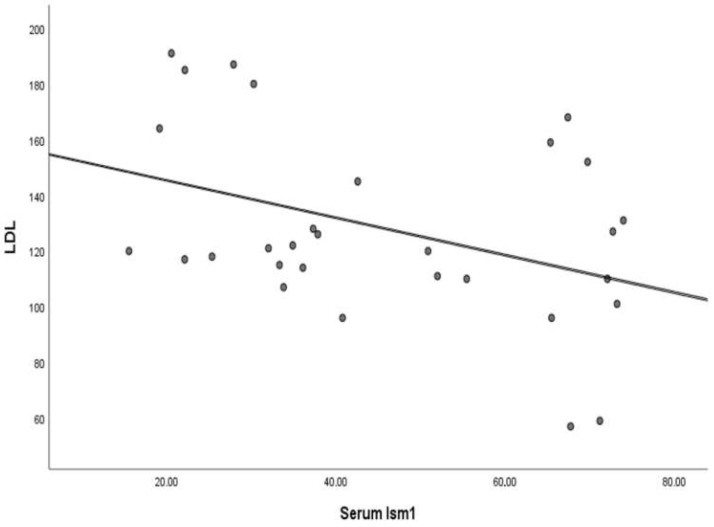
Scatterplot of the correlation between isthmin 1 and LDL levels after 3 months of metformin treatment.

When the increase in serum Ism1 levels and decrease in FPG, HbA1c and LDL levels with 3 months of metformin treatment were statistically significant, it was thought that there might be a relationship between these parameters. Spearman correlation analysis was performed to evaluate this possible relationship. The correlation was only detected between Ism1 and LDL levels. Ism1 level was significantly negatively correlated with LDL levels (rho = −0.362, *p* = 0.05) (Table 4, Figure 3).

## 4. Discussion

Isthmin-1 was identified only three years ago as actually being an adipokine [9]. Since the effects of adipokines on glucose and lipid metabolism are known, Ism1 has quickly become a frequently investigated adipokine [16,17,18,19]. This research differs from previous ones because Ism1 immunoreactivity in salivary glands was detected for the first time, suggesting its potential as a non-invasive follow-up indicator. Furthermore, Ism1 was found to be increased with metformin treatment in the newly diagnosed T2DM patients.

Glucose homeostasis is a continuous event, and the production of glucose is predominantly ensured by the liver [16]. On the other hand, studies have shown that adipose tissue can be considered an active endocrine gland since it can synthesize adipokines. These adipokines and their association with T2DM have been evaluated in many studies, especially in the last decade [20]. Asprosin, adiponectin, resistin, fatty acid binding protein 4 and leptin are just a few of these adipokines in these studies [21,22,23]. Ism1 is a much newer adipokine, and its effect on glucose metabolism has been experimentally evaluated before. Jiang et al. [10] observed that glucose uptake in adipocytes was severely reduced when they knock down the Ism1 protein. In the same study, Ism1 was said to be comparable to metformin in terms of its effect of increasing glucose tolerance in mice [10]. They demonstrate that Ism1 is a secreted polypeptide hormone that regulates adipose tissue glucose uptake while reducing steatosis in the liver. They experienced a series of cell culture experimental mouse studies at a more molecular and pathway-oriented level. Also, they administered Ism1 therapeutically in vivo. Their results were mostly discussed in comparison with insulin because Ism1 and insulin both utilize the PI3K-AKT signaling pathway to mediate their effects, as mentioned before. The most striking finding was that Ism1 reduces lipogenesis, whereas insulin increases it. With these results, the study by Jiang et al. [10] has been the most supportive and frequently referenced research in the discussion of our clinical findings. Here, in this clinical study, we found that Ism1 increased after the initiation of metformin treatment in diabetic patients. It can be interpreted that Ism1 is a drug-inducible adipokine. In the study by Wang et al. [13], Ism1 was found to be low in T2DM patients and high in control group participants. And Ism1 has been reported to be an independent protector in T2DM. In the literature, results have been found that Ism1 increases or decreases in diabetes. However, in both cases, Ism1 has been interpreted as an independent protective adipokine in diabetes due to its effects. In our study, it was found that Ism1 serum levels did not significantly change in diabetic patients. The significant increase in Ism1 levels after 3 months of treatment was attributed to metformin.

In the limited number of previous studies, it has been reported that Ism is mostly affected by factors such as age, gender, adolescence and BMI. Ruiz-Ojeda et al. [24] researched the Ism1 levels during adolescence. Also, increased levels of Ism1 were found in obese boys during adolescence. In this study, Ism1 levels may have been shaped by both obesity, gender and severe hormonal changes. If our results are interpreted according to this study, the fact that the majority of patients were male may have contributed to the increase in Ism1. In a very recent study, Ism1 levels were found to be significantly higher in men than in women, supporting our results [25]. On the other hand, the obesity of male adolescents may also have contributed to the increase in Ism1. A study mentioned above reported that Ism1 expression showed a positive and significant correlation with BMI too. In this study, individuals with BMI > 28 were grouped according to their weight [10]. BMI, the common factor of both studies, was not a grouping criterion in our study. In addition, BMI did not change among healthy participants and newly diagnosed diabetics (even after 3 months of metformin treatment). In a study emphasizing that Ism1 levels are higher in overweight/obese patients compared to lean patients, it was found that Ism showed an inverse correlation with age. Moreover, Ism1 was also found to be higher in T2DM compared to controls [26]. In the present study, the mean age of participants with T2DM was higher than that of control group participants. However, as in BMI, the possible relationship with age was not measured since we focused on evaluating the action of metformin and the effect of metformin on Ism1 levels in the planning of our study. In addition to its well-known efficacy in T2DM, metformin is clearly beneficial in many pathologic conditions. However, the limits of its therapeutic efficacy and the underlying mechanisms are varied and still unclear [27]. The significant increase in Ism1 after 3 months of treatment in the present study, while it was at the same level as the control at the time of diagnosis, suggested that Ism1 may be one of the unknown mechanisms mediating the effects of metformin [28].

FPG, HbA1c, LDL and TG levels are expected to decrease with metformin [29]. In our study, they also decreased. In this case, we thought that Ism1 may play a role in this effect. Although there was no exact linear relationship, we analyzed the serum Ism1 concentrations, which did not follow a normal distribution and included outliers, with FPG, HbA1c, LDL and TG concentrations using Spearman correlation. We found a correlation only between serum Ism1 and serum LDL concentrations. Jiang et al. [10] reported that the overexpression of Ism1 prevents hepatic steatosis in obese mice, which supports our findings. On the other hand, in a study with a different design compared to our study, patients with T2DM were divided into four groups according to serum HDL levels. In T2DM, serum Ism-1 and HDL were found to be negatively correlated [30]. Our findings revealed a negative correlation between increased Ism1 and decreased LDL levels following metformin treatment. Since the relationship between lipid parameters and Ism1 levels reported in the literature is not entirely consistent with the results of our study, the focus has been placed on the details of metformin. Metformin is known to reduce serum lipids by increasing the efficacy of a lipid-lowering drug such as atorvastatin or by monotherapy alone [27,31]. The pathway that mediates this effect most frequently is known to be the AMP-activated protein kinase pathway [32]. However, Zhao et al. [33] reported that the effects of Ism1 on metabolism are mediated by the phosphoinositide 3-kinase (PI3K)-Akt signaling pathway independently of insulin. When previous studies and the findings of our study are combined, it can be inferred that metformin increases Ism1 expression and that Ism1, independently of insulin, mediates the beneficial effects of metformin on lipid metabolism.

Increasing our knowledge about the underlying mechanisms and mediating molecules may lead to possible new therapeutic targets for T2DM or may ensure new diagnostic or follow-up tools/tests. HbA1c is used in the diagnosis of diabetes by giving an idea about the mean plasma glucose levels for the last 2–3 months. Furthermore, it is also included in the follow-up of diabetes regulation [34,35]. However, HbA1c is obtained with an invasive blood sampling. Moreover, adipokines associated with glucose homeostasis in biological fluids such as saliva, an alternative to blood samples, have been recognized to have diagnostic/follow-up potential [36]. Asprosin, leptin, adiponectin, resistin and visfatin are adipokines that have been detected in saliva in previous studies [37,38,39]. The desired result of detecting these adipokines in saliva tissue is to find a much easier, reproducible and noninvasive method than blood withdrawal, just as we thought in our study. We thought that Ism1 may be a non-invasive follow-up marker by only using an oral swap. Immunoreactivity of Ism1 in salivary glands was detected. However, the numerically increased levels of Ism1 in saliva did not reach statistical significance. Therefore, we could not confirm our hypothesis that Ism1 could be a noninvasive follow-up marker. It may not be because of the Ism1 adipokine. It may be entirely due to the difficulties of performing ELISA tests to measure adipokines in saliva. First of all, more sensitive measurements may be required to measure much lower concentrations in saliva than in blood. Various proteolytic enzymes and proteins in saliva may interfere with the accuracy of the assay and the binding of antibodies. Factors such as salivary hydration status and recent food intake may cause inconsistencies in measurements, making the standardization of ELISA tests difficult. The lack of universally accepted protocols for saliva collection, storage and processing, as well as the small sample size and the heterogeneity of our demographically and characteristically heterogeneous sample, may also have influenced the results. Despite these difficulties, the fact that adipokines were measurable in saliva in some studies (e.g., resistin, visfatin, TNF-α and ghrelin levels in unstimulated whole saliva in type 2 diabetes) may be related to the investigators’ use of specialized assay kits designed for saliva samples [40].

## 5. Conclusions

Although Ism1 immunoreactivity in the salivary glands was detected for the first time, no notable increase in Ism1 levels in saliva was detected. On the other hand, Ism1 was found to be increased with metformin treatment. Further analysis, which evaluated the relationships of parameters whose levels changed significantly with metformin, revealed an inverse correlation between Ism1 and LDL. Considering the entire study, the hypothesis that Ism1 could be a follow-up marker in T2DM could not be confirmed. However, the most striking finding of this study is that Ism1 may mediate the ameliorative effect of metformin, especially on serum LDL. Nonetheless, clinical studies with larger populations and well-planned animal experiments are still needed. With experimental and clinical studies that support and expand our results, Ism1 could become a new therapeutic and/or follow-up target in the future.

## Figures and Tables

**Table 1:** Demographic properties of participants.

		Control n (%)	New-Onset DM n (%)	*p* Value
age ^#^		42.10 ± 8.94	52.64 ± 9.47	0.004
sex	M	24 (80.0)	17 (56.7)	0.095
	F	6 (20.0)	13 (43.3)	

^#^ The mean ± sd was used as a descriptive.

**Table 2:** Change in biochemical parameters.

	Control	New-Onset DM	DM-Met	*p* Value *	*p* Value ^#^
	Median (Min–Max)	Median (Min–Max)	Median (Min–Max)		
FPG (mg/dL)	90.50 (64–109)	171.50 (108–303)	125.0 (88–285)	<0.001	<0.001
HbA1c (%)	6.0 (4–10.6)	7.3 (5.3–12.4)	6.44 (5.1–11.6)	<0.001	<0.001
BMI (kg/m^2^)	27.3 (26.8–29.2)	28.2 (27.3–34.1)	28.4 (27.0–33.4)	0.134	0.566
AST (IU/L)	18 (10–71)	20.50 (8–77)	20 (12–53)	0.325	0.380
ALT (IU/L)	19.50 (11–68)	25.50 (10–91)	22.50 (8–78)	0.027	0.106
Urea (mg/dL)	25.00 (12–56)	28.00 (15–57)	29.50 (17–73)	0.011	0.257
Cr (mg/dL)	0.70 (0.48–1.70)	0.66 (0.46–1.70)	0.70 (0.38–1.5)	0.173	0.951
LDL (mg/dL)	118.0 (57–191)	136.0 (64–325)	120.50 (60–185)	0.004	0.015
TG (mg/dL)	199.0 (53–376)	205.0 (83–747)	200.0 (55–369)	0.438	0.054

The data are expressed as the median, minimum and maximum values. DM-Met demonstrates the twelve weeks of metformin treatment. FPG: fasting plasma glucose, AST: aspartate aminotransferase, ALT: Alanin aminotransferase, Cr: creatinin, LDL: low-density lipoprotein, TG: triglyceride. *: *p* value, control to DM at diagnosis. ^#^: *p* value, DM to DM-Met.

**Table 3:** Serum and saliva levels of isthmin 1.

	Control	New-Onset DM	DM-Met	*p* Value *	*p* Value ^#^
	Median (Min–Max)	Median (Min–Max)	Median (Min–Max)		
Serum Ism1 (ng/mL)	25.70 (9.25–68.67)	25.95 (8.38–71.76)	39.30 (15.54–73.92)	0.923	0.028
Saliva Ism1 (ng/mL)	15.89 (2.67–37.81)	14.56 (3.09–44.98)	16.15 (7.59–26.63)	0.813	0.510

*: *p* value, control to DM at diagnosis. ^#^: *p* value, DM to DM-Met.

**Table 4:** Spearman correlation results.

Ism1		FPG	HbA1c	LDL
At diagnosis	rho	0.161	0.216	−0.204
	*p* value	0.396	0.252	0.279
3 months after metformin treatment	rho	−0.147	0.105	−0.362 *
	*p* value	0.437	0.580	0.050

FPG: Fasting plasma glucose; LDL: low-density lipoprotein. * Correlation is significant at the 0.05 level (2-tailed).

## Data Availability

The original contributions presented in this study are included in the article. Further inquiries can be directed to the corresponding author.
